# The metaproteome of the gut microbiota in pediatric patients affected by COVID-19

**DOI:** 10.3389/fcimb.2023.1327889

**Published:** 2023-12-22

**Authors:** Valeria Marzano, Stefano Levi Mortera, Chiara Marangelo, Antonia Piazzesi, Federica Rapisarda, Stefania Pane, Federica Del Chierico, Pamela Vernocchi, Lorenza Romani, Andrea Campana, Paolo Palma, Lorenza Putignani, the CACTUS Study Team

**Affiliations:** ^1^ Research Unit of Human Microbiome, Bambino Gesù Children’s Hospital, IRCCS, Rome, Italy; ^2^ Unit of Microbiomics, Bambino Gesù Children’s Hospital, IRCCS, Rome, Italy; ^3^ Unit of Infectious Disease, Bambino Gesu’ Children’s Hospital, IRCCS, Rome, Italy; ^4^ Department of Pediatrics, Bambino Gesù Children’s Hospital, IRCCS, Rome, Italy; ^5^ Research Unit of Clinical Immunology and Vaccinology, Bambino Gesù Children’s Hospital, IRCCS, Rome, Italy; ^6^ Unit of Microbiomics and Research Unit of Human Microbiome, Bambino Gesù Children’s Hospital, IRCCS, Rome, Italy

**Keywords:** COVID-19, gut microbiota, metaproteomics, mass spectrometry, functional patterns, pediatrics, SARS-CoV-2

## Abstract

**Introduction:**

The gut microbiota (GM) play a significant role in the infectivity and severity of COVID-19 infection. However, the available literature primarily focuses on adult patients and it is known that the microbiota undergoes changes throughout the lifespan, with significant alterations occurring during infancy and subsequently stabilizing during adulthood. Moreover, children have exhibited milder symptoms of COVID-19 disease, which has been associated with the abundance of certain protective bacteria. Here, we examine the metaproteome of pediatric patients to uncover the biological mechanisms that underlie this protective effect of the GM.

**Methods:**

We performed nanoliquid chromatography coupled with tandem mass spectrometry on a high resolution analytical platform, resulting in label free quantification of bacterial protein groups (PGs), along with functional annotations via COG and KEGG databases by MetaLab-MAG. Additionally, taxonomic assignment was possible through the use of the lowest common ancestor algorithm provided by Unipept software.

**Results:**

A COVID-19 GM functional dissimilarity respect to healthy subjects was identified by univariate analysis. The alteration in COVID-19 GM function is primarily based on bacterial pathways that predominantly involve metabolic processes, such as those related to tryptophan, butanoate, fatty acid, and bile acid biosynthesis, as well as antibiotic resistance and virulence.

**Discussion:**

These findings highlight the mechanisms by which the pediatric GM could contribute to protection against the more severe manifestations of the disease in children. Uncovering these mechanisms can, therefore, have important implications in the discovery of novel adjuvant therapies for severe COVID-19.

## 1 Introduction

Coronavirus disease 2019 (COVID-19), caused by severe acute respiratory syndrome coronavirus 2 (SARS-CoV-2), emerged in late 2019 (Zhu et al., 2020); in 2020 the World Health Organization (WHO) declared a global emergency due to the severe public health effects. To date, more than 700 million cases and approximately 7 million deaths due to COVID-19 have been confirmed around the world (https://covid19.who.int, accessed on 14 June 2023). It is well known that the respiratory system is the main target of SARS-CoV-2 infection. However, while respiratory symptoms are present in a major part of cases (Wu and McGoogan, 2020), gastrointestinal (GI) symptoms, such as diarrhea, nausea or vomiting, and abdominal pain (Alimohamadi et al., 2020) have been reported up to 20% in patients with COVID-19 (Cheung et al., 2020; Huang et al., 2020; Wu and McGoogan, 2020).

The process of virus infection in host cells occurs through the binding of the spike (S) protein of SARS-CoV-2 to the host receptor of the angiotensin-converting enzyme 2 (ACE2) (Medina-Barandica et al., 2023). ACE2 is also expressed in epithelial cells of the digestive, renal and skin tracts, indicating that each of these could be a potential target for the virus (Jamshidi et al., 2021). In particular, up to 50% of adults and 30% children affected with COVID-19 had SARS-CoV-2 positive stool samples (De Ioris et al., 2020; Zuo et al., 2020).

Additionally, SARS-CoV-2 RNA was identified in stools or rectal swabs from patients with COVID-19, even when the virus was no longer present in the respiratory tract (Wu et al., 2020; Xiao et al., 2020), leading to the hypothesis that, in the intestine, there is not only a replication and activity but a greater permanence of the virus as well (Donati Zeppa et al., 2020). SARS-CoV-2 infection reduces the expression of ACE2 in the GI tract and the number of circulating angiogenic cells (CACs), thus affecting the gut endothelium, and, hence, triggering intestinal dysbiosis (Chhibber-Goel et al., 2021). It is noteworthy that the number of ACE-2 receptors in the duodenum increase with age, suggesting a potential entry mechanism for the SARS-CoV-2 (Vuille-dit-Bille et al., 2020). The above observations implicate an important role of host gut in the infectivity and severity of COVID-19 infection (Yang et al., 2020). Moreover, GI inflammatory conditions modify the multilayer barrier system and increase the expression of ACE2 in the intestinal epithelium, allowing SARS-CoV-2 to enter the intestinal epithelial cells (Kumar et al., 2020). Furthermore, the viscous mucus of the GI tract protects viral RNA and viral particles, allowing the virus to maintain its infectivity (Zhang et al., 2021). However, the GI tract is populated by the gut microbiota (GM), which plays multiple functions, including the maintenance of the intestinal epithelial barrier integrity, host immunomodulation, and the protection against pathogens. Indeed, evidence suggests that there is a three-sided connection between the composition of the GM, the human genome and COVID-19 (Tong et al., 2023).

Seeking to decipher this relationship, in a previous work, we have described the GM ecology in pediatric patients affected by SARS-CoV-2 using a targeted-metagenomic approach (Romani et al., 2022). The results showed that, in these patients, the GM actually exerted anti-inflammatory properties, conferring an advantage, compared to adult subjects, in reducing or preventing severe disease. Indeed, most children with COVID-19 are asymptomatic or show mild symptoms, most typically fever, cough, pharyngitis, GI symptoms and alterations in olfaction or taste (Romani et al., 2020; Zachariah et al., 2020; Zimmermann and Curtis, 2020; Zimmermann and Curtis, 2022).

In the current study, a subset of the patients enrolled by Romani et al., was analyzed in term of GM metaproteome at the onset of the disease, and compared with an age- and sex-matched healthy subject group, chosen as controls (CTRLs).

The metaproteomic approach herein reported, allowed us to provide a functional rationale for some trends in bacterial distribution and related metaproteins of the GM, in order to further elucidate the mechanisms by which the GM can function as a protective force against more severe manifestations of COVID-19.

## 2 Subjects and methods

### 2.1 Sample collection

A cohort of pediatric patients, with COVID-19 symptoms and tested positive for a nasopharyngeal swab, were enrolled after acceptance at the Emergency Department of Bambino Gesù Children’s Hospital (OPBG) in Rome, Italy, between March 1 and September 30, 2020 (Romani et al., 2022). Patient information including age, sex, and both clinical and routine laboratory data were collected. Patients diagnosed with COVID-19 were categorized according to disease severity using the WHO clinical progression scale (Lamontagne et al., 2020): i) “mild” without evidence of viral pneumonia or hypoxia; ii) “moderate” in presence of non-severe pneumonia (cough or difficulty breathing, fast breathing, and/or chest indrawing), and iii) “severe” in presence of pneumonia (cough or difficulty breathing) and at least one of the following: a) central cyanosis or SpO2 < 90%, severe respiratory distress, general danger sign of inability to breastfeed or drink, lethargy, unconsciousness, or convulsions; b) fast breathing. Stool samples were collected within 48–72 h since admission and stored at − 80°C, until processing.

Samples of healthy CTRLs, who were matched by age and sex, were chosen from the Microbiome Biobank of OPBG (Italian node of the Biobanking and Bio Molecular Resources Research Infrastructure), and the criteria for inclusion were as follows: normal weight, no GI disorders, and no use of antibiotics or probiotics within one month prior to sampling. The CTRL cohort was recruited by an epidemiological survey, conducted by the OPBG Human Microbiome Unit, to study pediatric gut microbiota programming, in accordance with the recommendations of the OPBG Ethics Committee (Protocols code 1113_OPBG_2016 and 2839_OPBG_2022).

The study protocol was performed in accordance with the Principles of Good Clinical Practice and Declaration of Helsinki, and approved by OPBG Ethical Committee (Protocol code 2083_OPBG_2020). Written informed consent for this study was signed by parents or legal representatives of children.

### 2.2 Sample preparation

The procedures for extracting bacterial proteins from feces, protein lysis and digestion were slightly modified from Levi Mortera et al. (Levi Mortera et al., 2022). Accordingly, after being slowly thawed 300 mg of material were dissolved in 6 ml of ice-cold phosphate buffer (DPBS; 200 mg/L KCl, 200 mg/L KH_2_PO_4_, 8,000 mg/L NaCl, 1,150 mg/L Na_2_HPO_4_), with 1% Triton X-100, a nonionic detergent to inactivate any presence of SARS-CoV-2 (Patterson et al., 2020). The samples were rapidly vortexed, shaken for 10 min and the slurry centrifuged 15 min at 402 x *g* and 4°C. The supernatant was collected, while the pellet was suspended in 6 mL of ice-cold DPBS. The procedure was repeated twice. All supernatants from each sample were centrifuged two more times at 402 x *g* for 15 min, 4°C, to remove debris and were then transferred into centrifuge tubes (Beckman Coulter, 29 x104 mm, 50 mL). After centrifuging at 16,000 x *g*, for 15 min, 4°C, the bacteria cell pellet was obtained and washed three times with ice-cold DPBS. After lysis, 50 µg of proteins were digested with Sequencing grade Trypsin (Promega, Milan, Italy) by filter-aided sample preparation (FASP) protocol (Levi Mortera et al., 2022).

### 2.3 Mass spectrometry analysis

Obtained peptides were quantified by spectrophotometer measures (NanoDrop 2000, Thermo Fisher Scientific), using a standard curve of MassPrep *Escherichia coli* digestion (Waters, Milford, Massachusetts, USA), and analyzed by nanoLiquid Chromatography-ElectroSpray Ionisation-tandem mass spectrometry (nanoLC-ESI-MS/MS), which was conducted on UltiMate3000 RSLCnano system, coupled to an Orbitrap Fusion Tribrid mass spectrometer with a nanoESI source [EASY-Spray NG (Thermo Fisher Scientific, Waltham, MA, USA)]. Peptides (2 μg) from each sample were first trapped on a μ-Precolumn C18 PepMap100 (5 µm particle size, 100 Å pore size, 300 µm i.d. x 5mm length, Thermo Fisher Scientific) at 10 μL/min, 3 minutes, with an aqueous solution of 2% Acetonitrile (ACN) and 0.1% Trifluoroacetic acid. Then, peptide elution was performed with an EASY spray column (75 μm x 50 cm, 2 μm particle size, Thermo Fisher Scientific) at a flow rate of 250 nL/min using a linear gradient of increasing organic solvent starting from 95% eluent A [0.1% Formic acid (FA) in water] to 25% eluent B (99.9% ACN, 0.1% FA) in 143 min, and total LC-run of 190 min and temperature of 40°C.

Orbitrap detection was used for data acquisition in both full scan, with a resolution of 120 K, in a range between 375 and 1,500 *m/z*, and data-dependent MS/MS analysis with a resolution of 15 K with a 3 s cycle-time, during which most abundant multiple-charged (2^+^ – 7^+^) precursor ions were isolated for activation after Quadrupole isolation with a 1.6 *m/z* isolation window and dynamic exclusion enabled for 30 s. The normalized collision energy was optimized at 30% for high-energy collisional dissociation (HCD). MS and MS/MS Automatic gain control (AGC) targets were set to standard mode with a maximum injection time of 50 ms and automatic, respectively. The intensity threshold for MS/MS was set to 50,000. For internal calibration, a lock mass of the polydimethylcyclosiloxane (445.12003 *m/z*) was used.

Two replicates were produced for each sample.

### 2.4 Bioinformatic analysis and statistics

NanoLC-ESI-MS/MS data were analyzed by MetaLab-MAG desktop version 1.0.2 (Cheng et al., 2023) setting carbamidomethylation of cysteine as fixed modification, protein N-terminal acetylation and oxidation of methionine as variable modification, maximum two missed cleavages. Databank searching was performed versus the Unified Human Gastrointestinal Genome (UHGG) v2.0.1 (Almeida et al., 2021) also including host UniProtKB/Swiss-Prot database (*Homo sapiens*). Peptide-Metaproteomic Analysis to extract taxonomic information by the Lowest Common Ancestor (LCA) algorithm was performed by Unipept Desktop version 2.0.0 (Verschaffelt et al., 2021).

The bioinformatic pipeline was performed by R version 4.3.1 *ad hoc* scripts including gtools, dplyr, vegan, mixOmics and ecodist as main packages. The output files from MetaLab-MAG and Unipept were collapsed to create a final comprehensive matrix. In details, Protein Group (PG) matrix was filtered, considering only PGs with a number of detected razor peptides ≥ 2. Hence, intensities of PGs of the two technical replicates were averaged. After Label Free Quantitation (LFQ) intensities of the PGs’ log10-transformation, the matrix was reduced to those PGs identified in at least 50% of the whole sample set. Imputation of missing values was performed by the K-nearest neighbors’ method using a neighborhood of √samples. The final matrix included PGs and their corresponding LFQ intensities, which were associated with both functional annotations [*e.g*., Clusters of Orthologous Groups (COG) (Galperin et al., 2021) name and category; Kyoto Encyclopedia of Genes and Genomes (KEGG) (Kanehisa, 2019) pathways] and taxonomy based on LCA rank, with taxonomic assignment at all possible levels (Levi Mortera et al., 2022).

Multivariate Bray-Curtis β-diversity, Principal Component Analysis (PCA), Partial Least Squares-Discriminant Analysis (PLS-DA), were computed on the final LFQ intensities’ PG matrix. To test association between covariates and β-diversity, permutational multivariate analysis of variance (PERMANOVA, 9,999 permutations) was employed.

The hierarchical cluster analysis, visualized by a heat map, was based on the LFQ PGs intensity abundances applying a z-score transformation, computing the distance function by Pearson correlation and the linkage by Ward clustering method (R pheatmap package).

After conducting a Shapiro-Wilk test to investigate data distribution, univariate comparisons were performed using the *t*-test to identify differential individual PGs between the two groups based on the LFQ PGs intensity abundances. The values were filtered through log_10_(fold change) values ≥ 0.3 or ≤ -0.3, which represents the ratio of the mean intensity of COVID-19 PG to the mean intensity of CTRLs PG, and *p*-value ≤ 0.05. The Kruskal-Wallis test was utilized instead to establish whether there existed a statistically significant variation in the medians of more than two subgroups.

Differentially expressed KEGG pathways were computed taking into consideration the mean abundance of PGs intensity with the same KEGG pathways for each sample in each sample groups, applying a t-test or a Kruskal-Wallis test, and selecting terms with false discovery rate (FDR)-adjusted *p*-values (using Benjamini-Hochberg correction) of ≤ 0.05.

Spearman’s correlation was used to assess the correlation between clinical characteristics and intensities of KEGG pathways, which were determined by the mean abundance of PGs intensity with the same KEGG pathways for each sample. Only correlations that were statistically significant with FDR-adjusted *p*-values (using Benjamini-Hochberg correction) of ≤ 0.05 were chosen.

The Database for Annotation, Visualization and Integrated Discovery (DAVID) Bioinformatic Resource (https://david.ncifcrf.gov/home.jsp) (Sherman et al., 2022) was used for the identification of enriched processes from human PGs. The DAVID Functional Annotation Clustering provided a functional annotation tool to highlight the most relevant terms of DAVID Knowledgebase v2023q2 associated with the uploaded human PGs’ list and discover enriched functional-related terms.

## 3 Results

### 3.1 Subjects’ cohort and GM metaproteome’s identification

Metaproteomic profiling of the GM was performed to evaluate microbial and functional signatures of the GM from stool samples of 21 patients with COVID-19, ranging in age from 1 to 16 years (mean of 10 years ± 5 s.d, 10 males and 11 females, male sex frequency of 48%) and compared to the GM of 21 healthy subjects (CTRLs) of the same age-range (min 1 − max 16 years, mean of 10 years ± 4 years s.d., 10 males and 11 females) with an equal distribution of male and female individuals. The patients’ anthropometric, demographic, and clinical data were recorded at enrollment (**Table 1**; **Supplementary file 1**).

**Table 1:** Demographic and clinical features of COVID-19 patients.

Variable	Cases N	Mean ± s.d* or frequency
**Age (years)**	21	10 ± 5
**Female sex**	11	52%
**Symptoms**		
Fever	14	67%
Cough	7	33%
Shortness of breath	2	10%
Diarrhea	2	10%
**Disease severity**		
Asymptomatic	4	19%
Mild	15	71%
Moderate	2	10%
**Blood result**		
Lymphopenia	7	33%
C reactive protein (mg/dL)	21	0.66 ± 2.10
**Radiological results suggestive of viral pneumonia**	5	24%
**Antibiotic treatment**	3	14%
**Comorbidities or coinfection**	0	0%

*s.d., standard deviation.

No statistically significant difference in age distribution and gender frequency was observed by comparing COVID-19 and CTRL groups (non-parametric Mann-Whitney test, *p*-value = 0.8102 and *p*-value =1.000, respectively). Moreover, the binomial two-tailed test revealed no statistically significant difference in gender proportions within each group (COVID-19, *p*-value = 1.000; CTRLs, *p*-value = 1.000).

**Figure 1:**
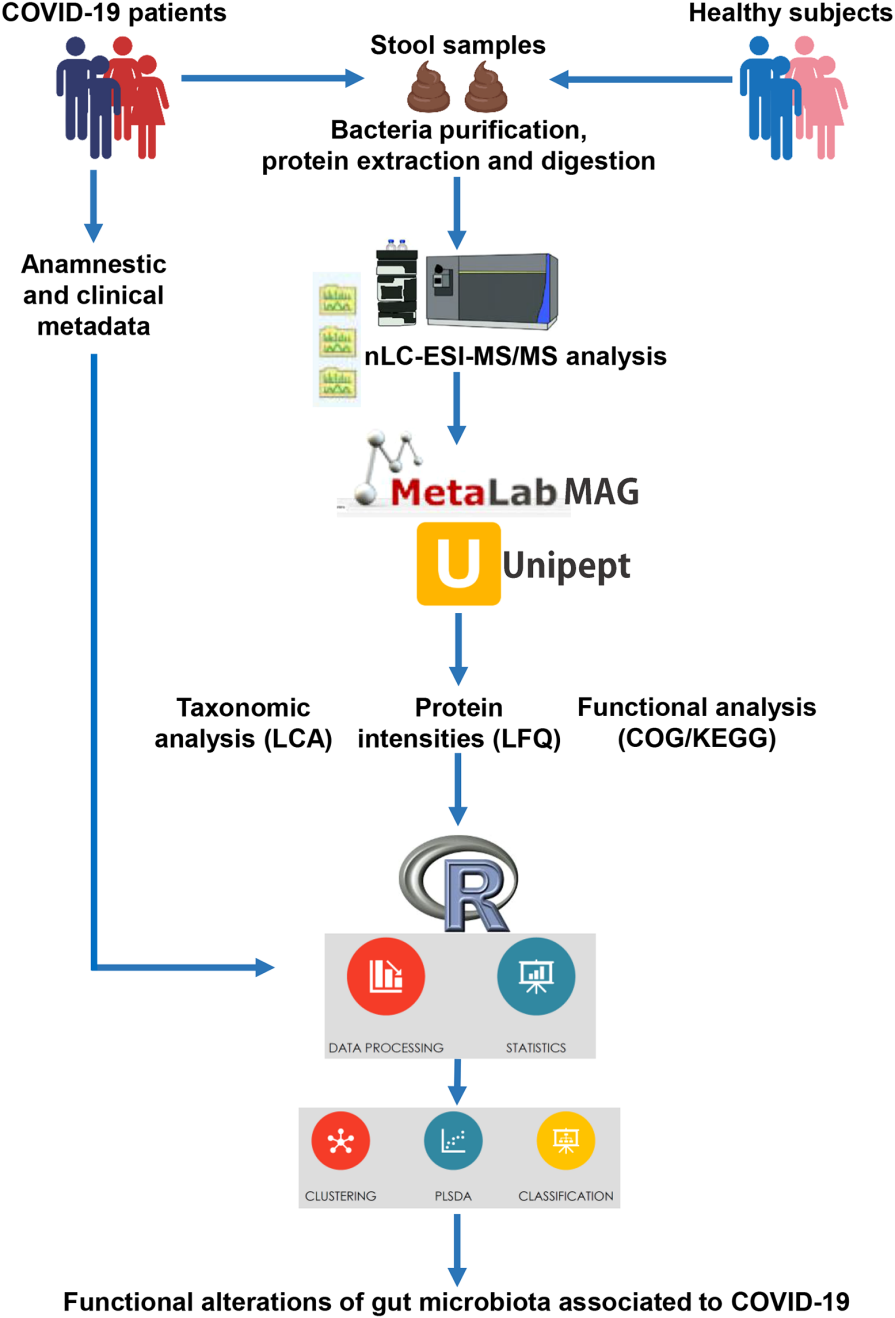
Illustration of the workflow applied for the metaproteomic analysis starting from faces of COVID-19 patients and age-matched healthy control subjects.

Fecal samples were subjected to the metaproteomic workflow to detect and quantify bacterial proteins, as illustrated in **Figure 1**.

Proteins from stool samples were extracted: 1,015 ± 576.7 µg (mean ± s.d.) and 1,021 ± 409.0 µg were obtained from patients and CTRLs, respectively (**Supplementary Figure 1A**). Upon enzymatic digestion of 50 µg of proteins, COVID-19 and CTRLs samples yielded 46.85 ± 13.58 µg and 52.69 ± 16.35 µg of peptides, respectively (**Supplementary Figure 1B**).

NanoLC-ESI-MS/MS procedure yielded a total of 48,735 PGs, along with 362,790 peptide sequences. On average, this procedure identified 31,280 ± 1,033 bacterial PGs and 509 ± 10 human PGs from each sample. After filtering out PGs that had fewer than 2 razor peptides, had common razor peptides across kingdom as determined by LCA algorithm through Unipept, and were identified in less than 50% of the entire sample set (Zhang et al., 2018), 34,422 PGs were retained. We then excluded PGs associated with viruses and archaea. As a result, we obtained a matrix consisting of 33,277 PGs of bacterial origin and 533 PGs of human origin.

### 3.2 Description of the GM metaproteome of COVID-19 patients

#### 3.2.1 Multivariate analysis

Beta-diversity analysis applied to the abundance distribution of PGs in COVID-19 and CTRL samples did not reveal any significant differences when applied to the entire PGs’ matrix (*p*-value = 0.151) or only to bacterial PGs (*p*-value = 0.189). However, human PGs indicated dissimilarity between the two groups (*p*-value = 0.03) (**Supplementary Figure 2A**).

A PCA analysis was conducted on the LFQ final matrix and exhibited a slight separation between COVID-19 and CTRLs groups (**Supplementary Figure 2B**). In addition, the PLS-DA algorithm indicated a slight separation between the two groups, with Variable Importance in Projection (VIP) scores ≥ 2 including 1,614 PGs (Q2 = 0.423, *p*-value = 0.11) (**Supplementary Figure 2C**). PLS-DA was also carried out on separate datasets, each including only bacterial PGs (Q2 = 0.419, *p*-value = 0.128), with the VIP ≥ 2 associated to 1,598 PGs, and human PGs (Q2 = 0.502, *p*-value = 0.01), with the VIP ≥ 2 including 18 PGs, respectively (**Supplementary Figure 2C**).

Cluster analysis based on the PGs’ LFQ intensity abundances resulted in a heat map that did not provide any clear clustering, indicating a lack of good similarity amongst samples within each COVID-19 and CTRL groups (**Supplementary Figure 3**).

#### 3.2.2 Univariate analysis

**Figure 2:**
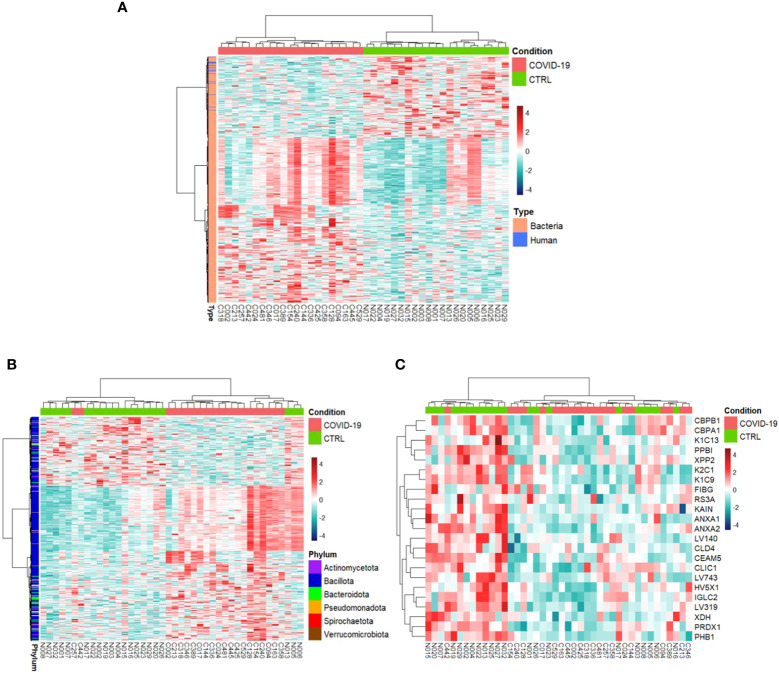
Hierarchical clustering of the differentially expressed protein groups (PGs). A heat map based on LFQ PGs’ intensity abundances and subject to a z-score transformation was used to visualize color-coded hierarchical cluster analysis from stool samples of patients (COVID-19, red color) and age-matched healthy subjects (CTRL, green color). The analysis was performed for all PGs (**A**, 698 differentially expressed PGs), bacterial PGs (675 differentially expressed PGs, **B**), and human PGs (23 differentially expressed PGs, **C**).

COVID-19s exhibited differential expression of 698 PGs in comparison to CTRLs (comprising of 675 bacterial PGs and 23 human PGs) (**Supplementary File 2**), whose clustering distinctly separates the two groups (**Figure 2**).

**Figure 3:**
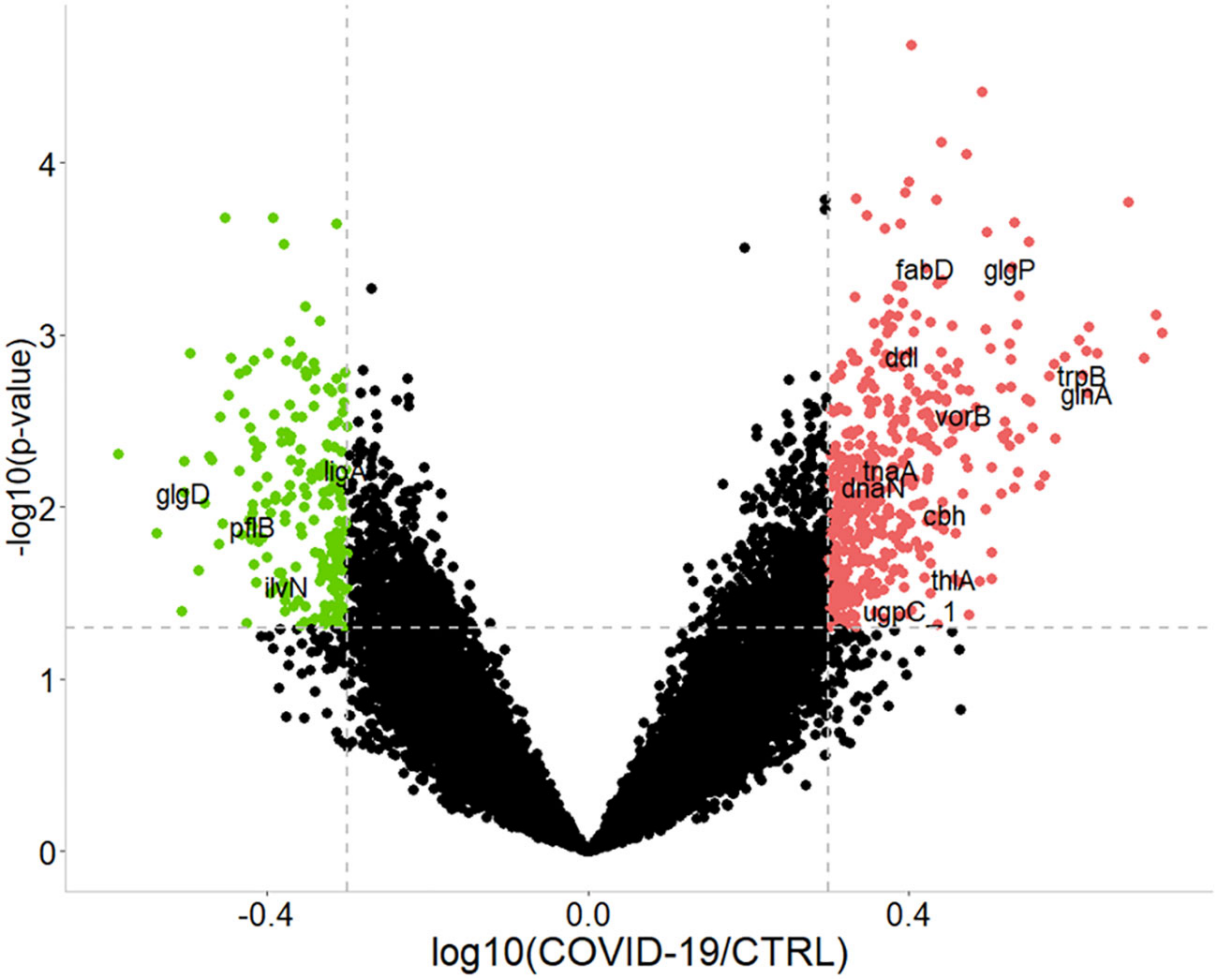
Graphical representation of the differentially expressed bacterial protein groups (PGs) comparing COVID-19 to CTRLs. Dashed gray lines indicate the set limits of log_10_(abundances’ ratio value) ≥ |0.3| and statistically significance values (–log_10_ *p*-value ≤ 0.05 established by *t*-test). Red and green circles indicate the changes for significant protein groups (over-expression and under-expression in COVID-19, respectively). Some PGs are highlighted by their acronym. For associations with the full name, protein group ID, as well as KEGG pathway and COG identifier, please refer to the Supplementary file 2 (*i.e*., tnaA, Tryptophanase 1, PG 3319, associated to tryptophan metabolism, COG3033).

Overall, 467 bacterial PGs were over-expressed and 208 were under-expressed in COVID-19 in respect of the CTRLs (**Figure 3**).

**Figure 4:**
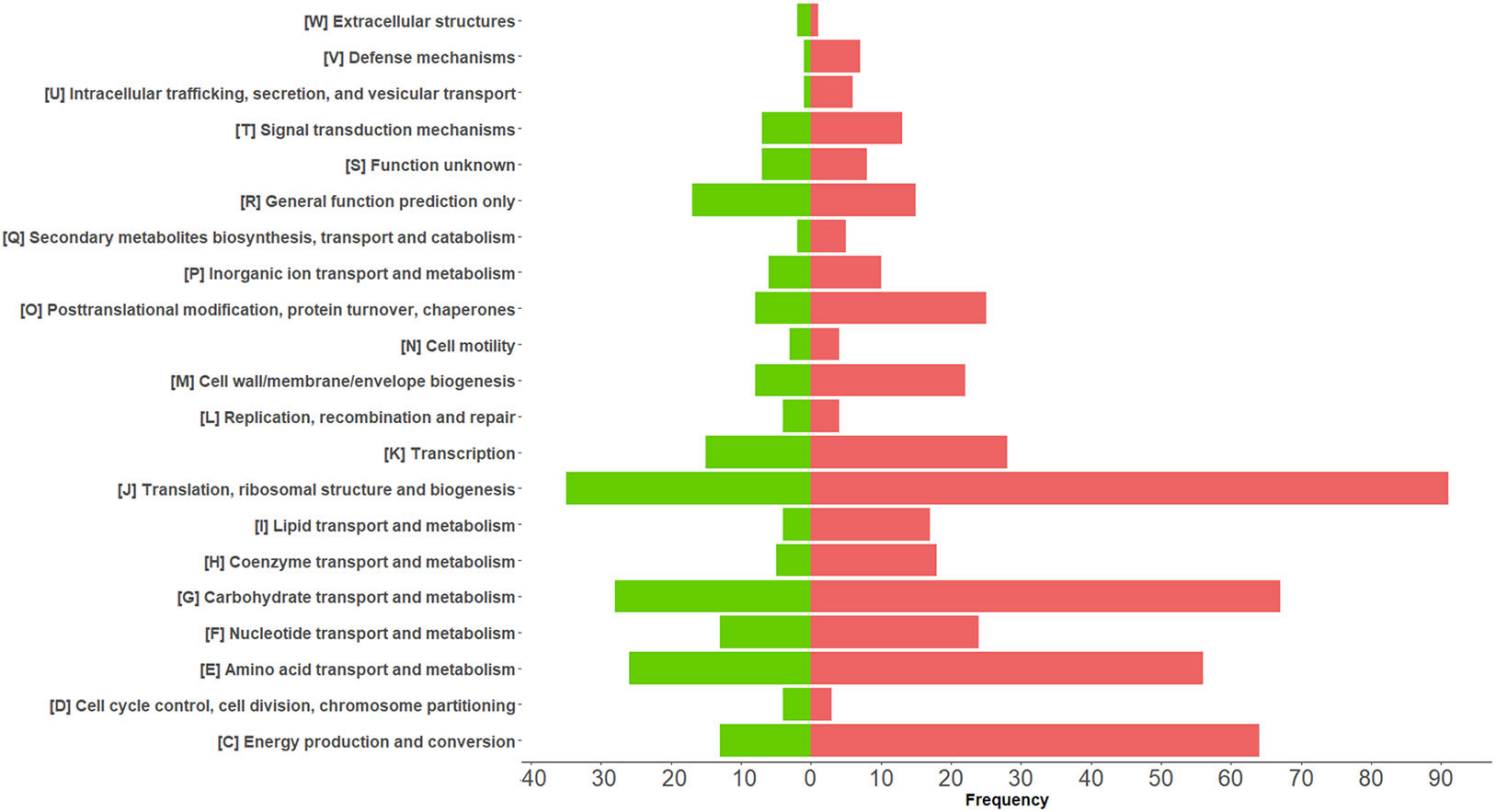
Graphical representation of COG categories modulated in differentially expressed bacterial protein groups (PGs) comparing COVID-19 with CTRLs. The bars represent the frequency of differentially expressed bacterial PGs associated with their functional annotation. Over-expressed and under-expressed PGs in COVID-19 are denoted by the color coding of red and green, respectively.

Among the 675 differentially abundant bacterial PGs, 633 had an assigned COG name, 411 a KEGG pathway name, and 645 a taxonomic assignment (**Supplementary File 2**), resulting in a total of 368 different COG names, 21 COG categories (**Figure 4**), and 114 KEGG pathways (**Supplementary Figure 4**). In addition, the LCA algorithm defined 108 unique taxa (**Table 2**).

**Table 2:** Association of differentially expressed bacteria protein groups to taxonomy.

Phylum (L2)	Distribution	N PGs			Family (L5)	N PGs			Genus (L6)	N PGs		
		total	over	under		total	over	under		total	over	Under
Actinomycetota	0.04%	25	10	15	Bifidobacteriaceae	16	5	11	*Bifidobacterium*	15	5	10
					Atopobiaceae	1	1		*Parolsenella*	1	1	
					Coriobacteriaceae	6	3	3	*Collinsella*	6	3	3
					Eggerthellaceae	1	1		*Gordonibacter*	1	1	
Bacillota	80.89%	546	391	155	Alicyclobacillaceae	1		1	*Tumebacillus*	1		1
					Paenibacillaceae	1	1		*Paenibacillus*	1	1	
					Streptococcaceae	3	2	1	*Streptococcus*	3	2	1
					Clostridiaceae	12	9	3	*Butyricicoccus*	1	1	
									*Clostridium*	11	8	3
					Eubacteriaceae	15	6	9	*Eubacterium*	15	6	9
					Eubacteriales Family XIII. Incertae Sedis	1	1					
					Heliobacteriaceae	1		1	*Heliobacterium*	1		1
					Lachnospiraceae	56	24	32	*Blautia*	5	1	4
									*Coprococcus*	13	11	2
									*Dorea*	2	1	1
									*Lachnoclostridium*	2	1	1
									*Lachnospira*	6		6
									*Mediterraneibacter*	3	1	2
									*Pseudobutyrivibrio*	1	1	
									*Roseburia*	9	1	8
					Oscillospiraceae	322	280	42	*Agathobaculum*	1		1
									*Anaeromassilibacillus*	2		2
									*Anaerotruncus*	1		1
									*Faecalibacterium*	58	48	10
									*Oscillibacter*	22	12	10
									*Pseudobacteroides*	1	1	
									*Pseudoflavonifractor*	1	1	
									*Ruminococcus*	219	213	6
									*Subdoligranulum*	15	3	12
					Peptostreptococcaceae	2	2		*Criibacterium*	1	1	
									*Peptostreptococcus*	1	1	
					Coprobacillaceae	4	2	2	*Catenibacterium*	4	2	2
					Erysipelotrichaceae	4	1	3	*Holdemania*	1	1	
									*Massilimicrobiota*	1		1
					Acidaminococcaceae	5	1	4	*Acidaminococcus*	1	1	
									*Phascolarctobacterium*	4		4
					Selenomonadaceae	5		5	*Megamonas*	5		5
					Veillonellaceae	13	6	7	*Dialister*	10	5	5
									*Megasphaera*	3	1	2
					Peptoniphilaceae	1	1		*Anaerosphaera*	1	1	
Bacteroidota	9.04%	61	39	22	Bacteroidaceae	10	7	3	*Bacteroides*	10	7	3
					Porphyromonadaceae	1	1					
					Prevotellaceae	18	7	11	*Paraprevotella*	1		1
									*Prevotella*	17	7	10
					Rikenellaceae	1	1		*Alistipes*	1	1	
					Tannerellaceae	1		1	*Parabacteroides*	1		1
					Prolixibacteraceae	1	1					
Pseudomonadota	1.48%	10	5	5	Caulobacteraceae	1		1	*Phenylobacterium*	1		1
					Alcaligenaceae	1	1		*Bordetella*	1	1	
					Shewanellaceae	1		1	*Shewanella*	1		1
					Halomonadaceae	3	2	1	*Halomonas*	3	2	1
					Piscirickettsiaceae	1		1	*Methylophaga*	1		1
Spirochaetota	0.15%	1	1		Treponemataceae	1	1		*Treponema*	1	1	
Verrucomicrobiota	0.15%	1	1		Akkermansiaceae	1	1		*Akkermansia*	1	1	

Five hundred and forty-six PGs (80.89%) of the differentially expressed bacterial PGs were assigned to the Bacillota phylum (formerly Firmicutes), with Oscillospiraceae (322 PGs), Lachnospiraceae (56 PGs), Eubacteriaceae (15 PGs), Veillonellaceae (13 PGs), and Clostridiaceae (12 PGs) families being prevalent. Actinomycetota or Actinobacteria accounted for 0.04% of the differentially expressed PGs (25 PGs), while Bacteroidota or Bacteroidetes accounted for 9.04% (61 PGs), Pseudomonadota (formerly Proteobacteria) accounted for 1.48% (10 PGs), and Spirochaetota or Spirochaetes and Verrucomicrobiota (formerly Verrucomicrobia), each accounted for 0.15% (1 PGs).

Considering the total number of PGs and their expression level, the most significant variations in the number of over- and under-expressed PGs were identified in the Bacillota phylum (391 over-expressed PGs *vs.* 155 under-expressed in COVID-19s), with special reference to the Oscillospiraceae family (280 over- vs. 42 under-expressed), and to the *Faecalibacterium* (48 over- vs. 10 under-expressed) and *Ruminococcus* (213 over- vs. 6 under-expressed) genera. Also, the Clostridiaceae family (9 vs. 3 PGs) and the *Clostridium* genus (8 *vs.* 3 PGs) showed a different regulation. Regarding the Actinomycetota phylum, the most important differences were observed for the Bifidobacteriaceae family (5 vs. 11 PGs) and for the *Bifidobacterium* genus (5 *vs.* 10 PGs). Lastly, Bacteroidota phylum showed remarkable difference for the Bacteroidaceae family and the *Bacteroides* genus (7 *vs.* 3 PGs).

**Figure 5:**
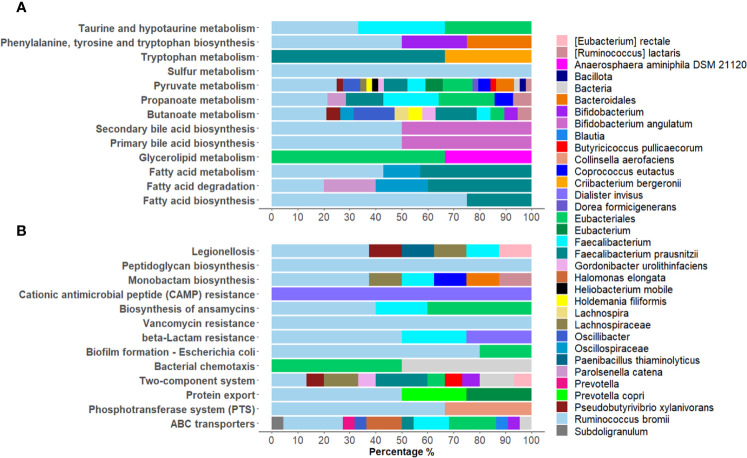
Graphical representation of the distribution of Lowest Common Ancestor (LCA) taxa within statistically over-expressed KEGG pathways in COVID-19. A selection of KEGG pathways belonging to metabolic pathways were displayed **(A)**, as well as processes of bacterial antibiotic resistance and virulence **(B)**.

Among the KEGG pathways associated with the differentially expressed bacterial PGs, statistically significant differences were found for 84 pathways, with 66 being over-expressed in COVID-19 group (**Supplementary Figure 5**). Among the others, the KEGGs were mostly associated with processes involving metabolic pathways as with antibiotic resistance and virulence. For the first group, 13 pathways were examined, in particular Butanoate metabolism; Fatty acid biosynthesis; Fatty acid degradation; Fatty acid metabolism; Glycerolipid metabolism; Phenylalanine, tyrosine and tryptophan biosynthesis; Primary bile acid biosynthesis; Propanoate metabolism; Pyruvate metabolism; Secondary bile acid biosynthesis; Sulfur metabolism; Taurine and hypotaurine metabolism; Tryptophan metabolism (**Figure 5A**). These pathways originated from 111 PGs, encompassing 32 distinct COG names among 8 COGs categories. The five most heavily populated categories were: Energy production and conversion [C] (55 PGs), Lipid transport and metabolism [I] (28 PGs), Carbohydrate transport and metabolism [G] (12 PGs), Amino Acid transport and metabolism [E] (9 PGs), Secondary metabolites biosyntesis, transport and catabolism [Q] (5 PGs).

Considering the taxonomic assignment, these 111 PGs were mainly associated with Bacillota (98 PGs, with 32% of them associated with *Ruminococcus* and 24% with *Faecalibacterium* genera) and Actinomycetota (8 PGs, with 50% of them belonging to the *Bifidobacterium* genus).

Within the second group of antibiotic resistance and virulence over-expressed KEGG pathways, 13 were reported, namely ABC transporters; Bacterial chemotaxis; β-Lactam resistance; Biofilm formation - *Escherichia coli*; Biosynthesis of ansamycins; Cationic antimicrobial peptide (CAMP) resistance; Legionellosis; Monobactam biosynthesis; Peptidoglycan biosynthesis; Phosphotransferase system (PTS); Protein export, Two-component system; and Vancomycin resistance (**Figure 5B**). These pathways corresponded to 81 PGs, with 32 different COG names across 13 COG categories. Amongst categories, the top five most heavily populated were Carbohydrate transport and metabolism [G] (27 PGs); Amino Acid transport and metabolism [E] (21 PGs); Cell wall/membrane/envelop biogenesis [M] (13 PGs); Energy production and conversion [C] (5 PGs); Cell motility [N] (5 PGs); and Intracellular trafficking, secretion and vesicular transport (5 PGs).

Linking these PGs to LCA taxonomy assignment, the phyla that contained the highest quantity of PGs resulted Bacillota (67 PGs, 45% of them associated to *Ruminococcus* and 15% to *Faecalibacterium*) and Actinomycetota (4 PGs, 50% of them *Bifidobacterium* genus) (**Figure 5B**).

Moreover, 3 differentially expressed pathways were associated with DNA repair and recombination systems. Both bacterial Nucleotide excision repair (NER) and Base excision repair (BER) KEGG pathways were detected in COVID-19 samples as under-expressed KEGG pathways (Benjamini-Hochberg adjusted *p*-value = 0.01 in both cases); while the Homologous recombination pathway was over-expressed (Benjamini-Hochberg adjusted *p*-value = 4.67 x 10^-04^).

Regarding human PGs, 23 were identified as differentially expressed, and all exhibited under-expression for COVID-19 set. Seventeen PGs had a matching KEGG name and were able to be linked to a total of 23 KEGG pathways. Carboxypeptidase A1 (P15085, CPA1), Carboxypeptidase B1 (P15086, CPB1), were mapped to Pancreatic secretion pathway; both PGs besides X-prolyl aminopeptidase 2 (O43895, XPNPEP2) were related to Protein digestion and absorption pathway. The Fibrinogen γ-chain (P02679, FGG), constituent of the coagulation pathways, was related to numerous KEGG pathways, including Complement and coagulation cascades; Neutrophil extracellular trap formation; Platelet activation; and Coronavirus disease. Moreover, 5 PGs were identified as regions of Immunoglobulins (4 regions of light chains and 1 region of heavy chains). As a consequence, the DAVID enrichment analysis yielded as primary term a cluster of Immunity and Adaptive Immunity UniProt biological processes (**Supplementary File 3**).

### 3.3 Description of the GM metaproteome of COVID-19 patients in relation to clinical features

Because three out of 21 patients (14%) received antibiotic treatment prior to fecal sample collection, two of them at admission time, within a window of 24 hours, and only one after a completed one weeks’ cycle, we assessed that this treatment did not affected overall the GM metaproteome of the gut microbiota. Indeed, no statistically significant differences were found between patients administered with antibiotics by β-diversity analysis (Bray-Curtis algorithm) when applied either to the entire PGs matrix (*p*-value = 0.1), or to the only bacterial or human PGs (*p*-value = 0.103 and 0.825, respectively) (**Supplementary Figure 6**). Therefore, the analysis of the COVID-19 cohort continued as a whole, without the exclusion of these three patients.

To examine the effect of patients’ clinical features (**Table 1**; **Supplementary File 1**) on the GM metaproteome, we evaluated the LFQ PGs distribution based on COVID-19 severity. We divided the sample set into asymptomatic, mild, and moderate disease categories. Nineteen percent of cases were asymptomatic, 71% were classified as “mild” and 10% had a “moderate” disease. None were classified as “severe”.

**Figure 6:**
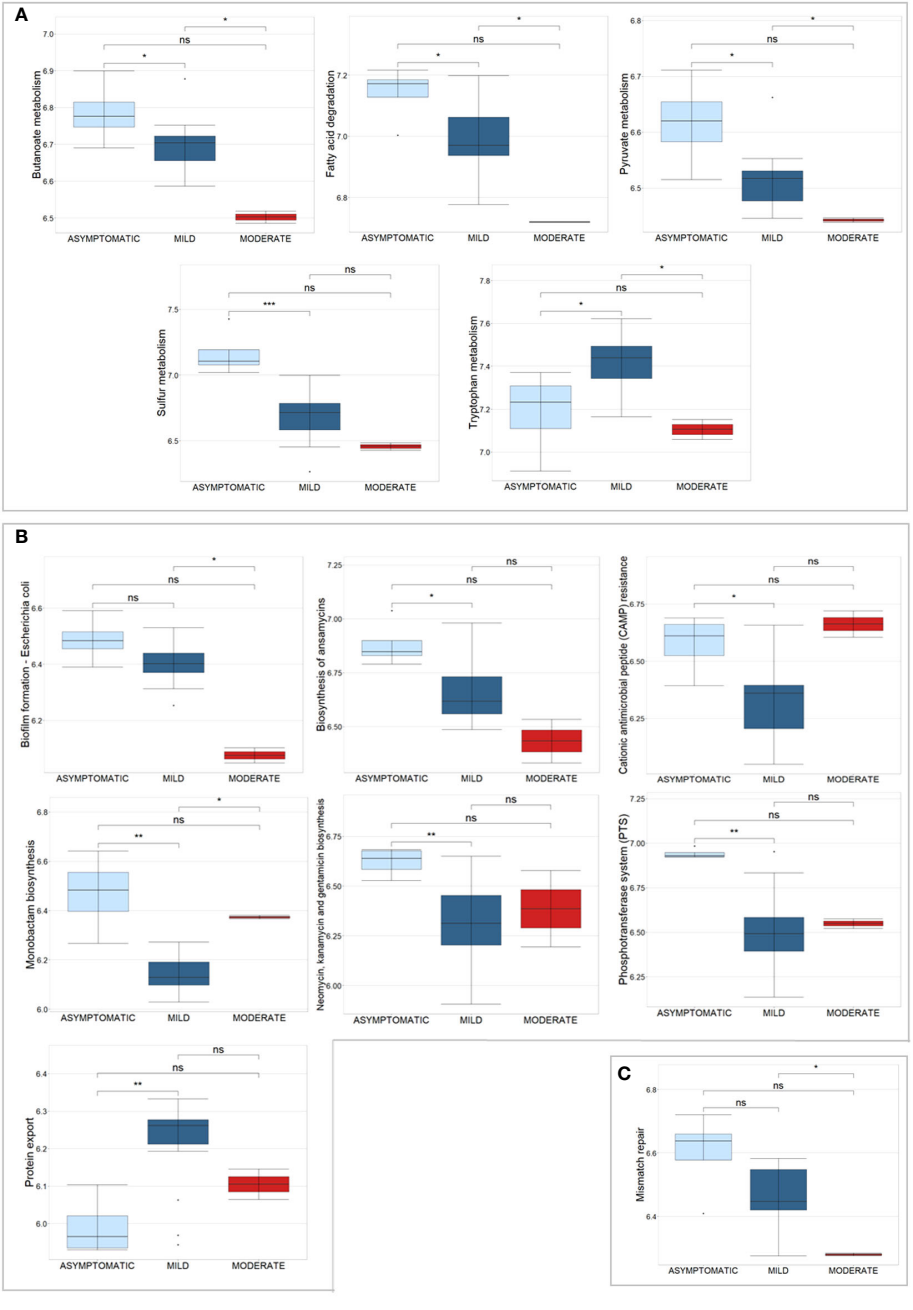
Box plots of a selection of statistically significant differential KEGG pathways in COVID-19 patients, classified according to disease severity, based on Kruskal-Wallis test. **(A)** displays KEGG pathways related to metabolic pathways, **(B)** shows antibiotic resistance and virulence processes, and **(C)** shows the Mismatch repair pathway. Significance of pair-wise comparisons (Mann-Whitney test, Benjamini-Hochberg adjusted *p*-value) were displayed: * *p*-value ≤ 0.05; ** *p*-value ≤ 0.01; *** *p*-value ≤ 0.001; and ns, no statistical significance (*p*-value > 0.05).

Univariate analysis revealed 1,181 bacterial and 6 human PGs differentially associated to the 3 subgroups, based on Kruskal-Wallis test comparison (**Supplementary File 4**). Of these bacterial PGs, 767 were associated with at least one KEGG pathway name, with a total of 117 different KEGG pathways identified. Among these, statistically significant differences were found for 63 pathways by computing a Krustal-Wallis test comparison, with a Benjamini-Hochberg adjusted *p*-value (**Supplementary File 4**). We decided to look more closely at the KEGG pathways that had already been identified as noteworthy in the comparisons between patients and CTRLs, *i.e.* metabolic pathways, antibiotic resistance and virulence processes, and DNA repair and recombination systems (**Figure 6**).

For the metabolism, 5 pathways were examined, in particular Butanoate metabolism (44 PGs), Fatty acid degradation (11 PGs), Pyruvate metabolism (59 PGs), Sulfur metabolism (5 PGs), and Tryptophan metabolism (3 PGs) (**Figure 6A**). These pathways originated from 122 PGs, encompassing 30 distinct COG names among 7 COGs categories. The four most heavily populated categories were: Energy production and conversion [C] (80 PGs), Lipid transport and metabolism [I] (18 PGs), Carbohydrate transport and metabolism [G] (14 PGs), and Amino Acid transport and metabolism [E] (8 PGs). Considering the taxonomic assignment, these 122 PGs were mainly associated with Bacillota (92 PGs, with 13% of them associated with *Eubacterium* and 13% with *Ruminococcus* genus) and Actinomycetota (9 PGs, with 78% of them belonging to the *Bifidobacterium* genus).

Within the second group of antibiotic resistance and virulence KEGG pathways, 7 were analyzed, namely Biofilm formation - *Escherichia coli*, Biosynthesis of ansamycins, Cationic antimicrobial peptide (CAMP) resistance, Monobactam biosynthesis, Neomycin, kanamycin and gentamicin biosynthesis, Phosphotransferase system (PTS), and Protein export (**Figure 6B**). These pathways corresponded to 60 PGs, with 27 different COG names across 7 COG categories. Amongst categories, the four most heavily populated were Carbohydrate transport and metabolism [G] (41 PGs); Amino Acid transport and metabolism [E] (9 PGs); Intracellular trafficking, secretion, and vesicular transport [U] (7 PGs), and Cell wall/membrane/envelop biogenesis [M] (6 PGs). Considering the taxonomic assignment, these 60 PGs were mainly associated with Bacillota (49 PGs, with 10% of them associated with *Faecalibacterium* and 10% with *Ruminococcus* genera) and Actinomycetota (8 PGs, with 63% of them belonging to the *Collinsella* genus, *Collinsella aerofaciens* specie).

The Mismatch repair KEGG pathway was identified by nine bacterial PGs, all of which belong to the Replication, recombination, and repair [L] COG category and are associated with the Bacillota phylum (**Figure 6C**). Specifically, 78% of them were of the Eubacteriales order; two were of the *Faecalibacterium prausnitzii* genus and one was of the *Roseburia faecis* genus.

Six human PGs were identified as differentially expressed across the three subsets based on COVID-19 severity: Cytokeratin-4, ADP-ribosylation factor 4, Ig lambda chain V-I region HA, Protein S100-P, Sodium/glucose cotransporter 1, and Ig mu chain C region (**Supplementary File 4**).

**Figure 7:**
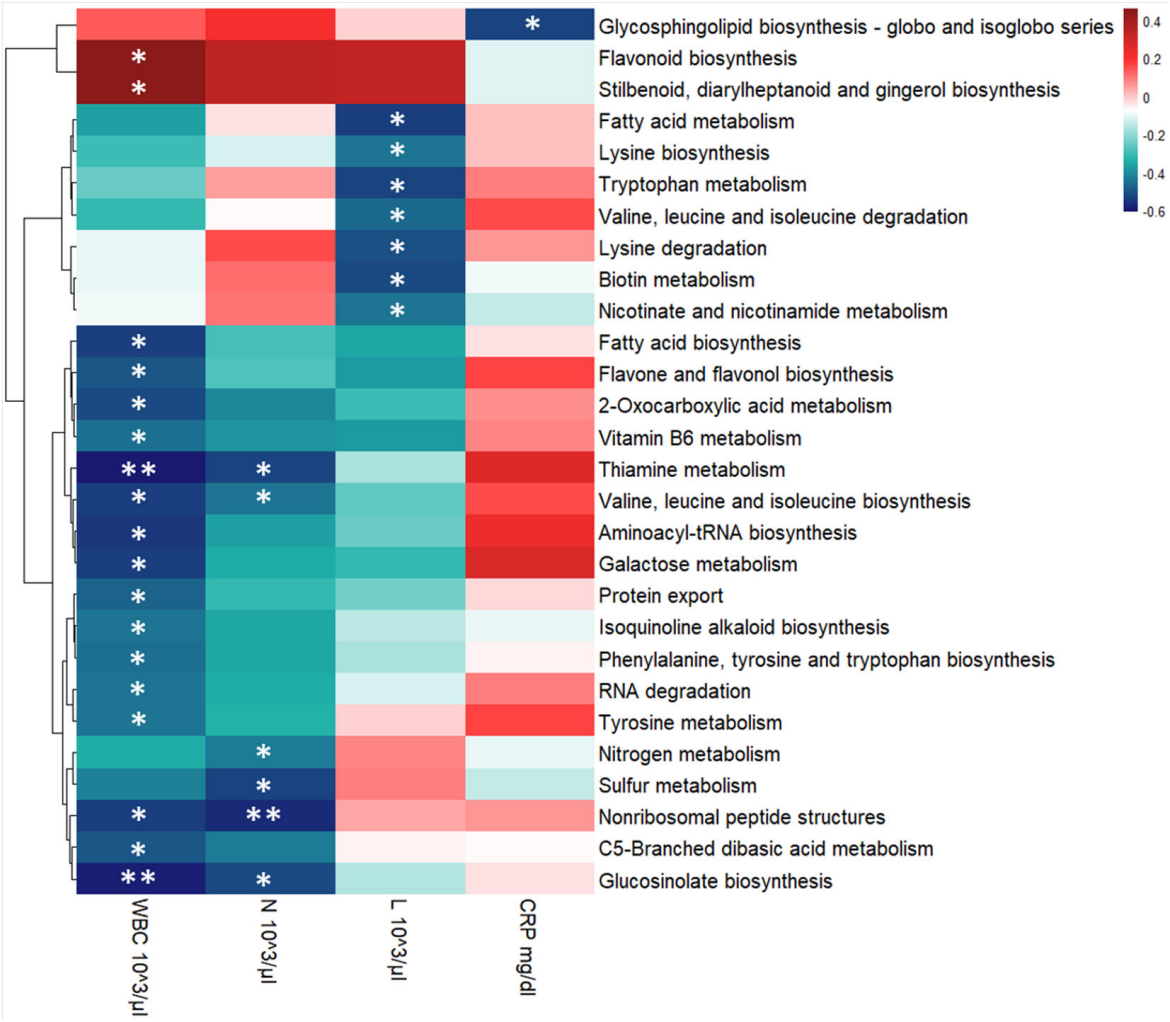
Heatmap showing Spearman’s correlations between KEGG pathways’ intensity and blood clinical features for the entire COVID-19 cohort. Red boxes indicate positive correlations, blue boxes show negative correlations. Benjamini-Hochberg adjusted *p*-value were displayed: * *p*-value ≤ 0.05 and ** *p*-value ≤ 0.01. KEGG pathways’ clusterization was computed by Euclidean distance. WBC, White Blood Cells; N, Neutrophils, L, Lymphocytes, and CRP, C-reactive protein.

To evaluate the relationship between hematological values, KEGG pathways and the GM metaproteome, we performed a correlation analysis. The majority of correlations were associated with the count of White Blood Cells (WBC), with 19 out of 21 KEGG pathways showing a negative correlation. In particular, the Lymphocyte (L) level displayed a negative correlation with 8 KEGG pathways (among them Biotin metabolism, Fatty acid metabolism, Lysine biosynthesis, Lysine degradation, Nicotinate and nicotinamide metabolism, Tryptophan metabolism, Valine, leucine and isoleucine degradation), while Neutrophils (N) count was associated to 6 KEGG pathways with a negative correlation (Glucosinolate biosynthesis, Nitrogen metabolism, Nonribosomal peptide structures, Sulfur metabolism, Thiamine metabolism, Valine, leucine and isoleucine biosynthesis) (**Figure 7**). Statistically significant negative correlations with C-reactive protein (CRP) level were found for only 2 KEGG pathways (Glycosphingolipid biosynthesis - globo and isoglobo series being one of them) (**Figure 7**).

## 4 Discussion

The gut contains numerous commensal species that perform crucial biological functions essential to human survival (Maciel-Fiuza et al., 2023). Additionally, these species inhabit a carefully balanced ecosystem. When this balance is upset, it can result in adverse effects on human health, a condition known as dysbiosis (Piazzesi and Putignani, 2022). Recently, there has been a growing interest in understanding how the non-bacterial components of the gut microbiota shape host physiology and immunity (Iorio et al., 2022). In this scenario, any perturbation, such as the emergence of a new virus that was not previously present, has implications for human health. Therefore, we decided to study how the presence of SARS-CoV-2 can modify the bacteriome - defined here as the GM - of pediatric patients by both a 16S rRNA-based metagenomics approach in a previous paper (Romani et al., 2022) and by a metaproteomic approach in the present study. Interactions among the host, microbiome members, and invading pathogens are complex and multidimensional, encompassing a wide range of organisms and variable factors. Research on these intricate interactions has been spurred by the availability of high-throughput metagenomic sequencing methods. However, it is important to note that certain predicted protein-coding genes derived from metagenomic data may not be expressed in a particular environment. Thus, relying solely on DNA sequencing data to predict the functional activities and interactions within microbial communities can lead to incomplete predictions. To better interpret these processes, it is necessary to study the abundance of transcripts, proteins, or metabolites (Salvato et al., 2021). Indeed, the metaproteomic approach allows us to understand the functional roles and interactions of the single components in the microbial community (Karaduta et al., 2021), therefore enriching the potential expression information acquired by metagenomics (Van Den Bossche et al., 2021). Hence, we chose to examine the GM of COVID-19 patients enlisted by Romani et al. also from a metaproteomic perspective. To our knowledge, this is the first metaproteomics study focused solely on pediatric patients. Only two previous metaproteomic studies have included pediatric cases in a larger cohort with varying ages (He et al., 2021; Sun et al., 2022), specifically 6 children out of 13 and 7 out of 63 individuals, respectively.

Another noteworthy aspect of the study presented in this paper is the young age of the subjects involved, specifically pediatric patients. Indeed, the microbiome undergoes modulation from birth to senescence, experiencing significant changes during early life, specifically during 1-3 years and after 3 years onwards (Stewart et al., 2018; Hill et al., 2021), and appearing to stabilize in healthy adults (Putignani et al., 2014; Lynch and Pedersen, 2016). So it is not possible to extrapolate GM data collected from a group of adult patients to children. Moreover, SARS-CoV-2 infection, in terms of disease severity, hospitalization and mortality, is a function of age (Zimmermann and Curtis, 2020; Zimmermann and Curtis, 2021). Therefore, our study may hold significance for further research in the field and may explain both the impact of SARS-CoV-2 virus on the GM and the differences in pathogenesis and clinical presentation between children and adults.

Our data demonstrated how SARS-CoV-2 infection affects the functionality of the GM at multiple levels. Specifically, it modifies metabolic pathways, mechanisms of antibiotic resistance and bacterial virulence, and DNA repair and recombination systems. Among the over-expressed metabolic pathways, those related to Phenylalanine, tyrosine and tryptophan biosynthesis and Tryptophan metabolism (**Figure 5**) may be a consequence also of the virus-dependent ACE2 reduction, since ACE2 is involved in tryptophan uptake (Zhou et al., 2022) acting as a chaperone of the amino acid transporter, B0AT1 in the small intestine (Takeshita and Yamamoto, 2022). The relationship among GM, host and ACE2 modulation by SARS-CoV-2 in modifying tryptophan pathways is complex: GM ecology is altered by a reduction in tryptophan absorption in ACE2 knockout mice, which in turn causes modification of tryptophan metabolites released by gut bacteria, which ultimately has an important impact on the host inflammatory response (Hashimoto et al., 2012; Zelante et al., 2013). The significance of ACE2 in gut function and biology is paramount also because it regulates the expression of antimicrobial peptides (Yu et al., 2021). Moreover, impaired tryptophan metabolism, resulting from decreased expression of corresponding genes, has been associated with critical SARS-CoV-2 infection compared to non-critical cases. It has been suggested that supplementing tryptophan or improving the tryptophan metabolic pathway could potentially mitigate severe COVID-19 outcomes (Yokoyama et al., 2022). In addition, tryptophanase 1, an enzyme that catalyzes the production of indole and pyruvate from L-tryptophan, was identified as one of the differentially expressed PGs. Tryptophan catabolites have been found to impact various physiological processes, which contributes to maintaining intestinal and systemic homeostasis in both human health and disease (Roager and Licht, 2018). Indeed, these metabolites activate the immune system by binding to the aryl hydrocarbon receptor, improve the intestinal epithelial barrier, stimulate gastrointestinal motility and hormone secretion, and exert varied effects such as anti-inflammatory, antioxidative, or toxic in the systemic circulation. As example, indole has a dual role, as it can both exhibit anti-inflammatory properties and exert a toxic effect due to one of its derivatives, indoxyl sulfate, which can cause nephrotoxicity and cardiovascular toxicity (Ye et al., 2022). Altogether, our results may show how the GM response may contribute to render the infection less severe in pediatric COVID-19 patients. Also the increased expression of sulfur metabolism in COVID-19 compared to CTRLs, as well as the decreasing trend of the associated PG observed by stratifying COVID-19 patients from asymptomatic to mild and severe infection, suggest an adaptive mechanism of the GM explaining the superior ability of pediatric GM to counteract the virus. Indeed, reactive sulfur compounds have demonstrated anti-inflammatory, antioxidant, and antiviral effects and have piqued interest for the treatment and prevention of the adverse effects diseases caused by SARS-CoV-2 (Iciek et al., 2022).

Regarding over-expressed bile acid pathways, a decrease in fecal secondary bile acid concentrations was found to be correlated with fatal outcomes of SARS-CoV-2 infection in adult patients (Stutz et al., 2022). Based on the findings of Stunz et al., it can be inferred that the increased expression of bile acid metabolism in our patients, as opposed to the CTRLs, signified the response of the GM, which has a positive effect on the host physiology and immune defense to overcome the infection. Indeed secondary bile acids affect differentiation of CD4 Th17 and Treg cells (Stutz et al., 2022). A similar hypothesis may be associated to the over-expression of Butanoate and Fatty acid metabolisms; indeed, short chain fatty acids (SCFAs, such as acetate, propionate and butyrate) family of compounds has recognized immunomodulatory and anti-inflammatory properties (Włodarczyk et al., 2022).

The over-expression of pathways related to antibiotic resistance and bacteria virulence may be viewed as a reaction to the SARS-CoV-2 virus. Indeed, bacteria modify their functions in response to interactions with the external environment. In a metagenomics study published by our collaborators, the PICRUSt algorithm identified predicted functional signatures mainly associated with bacterial virulence and antibiotic resistance (Laterza et al., 2023). It was found that KEGG orthologs modulated by the virus belonged to the two-component system, ATP-binding cassette proteins, and the PTS system, which were similar to those identified in our study. Regarding the involvement of ABC transporters, they have also been described, by metagenome and metatranscriptome sequencing, as in active expression in GM of COVID-19 patients along with beta-lactam resistance metabolic pathways (Zhou et al., 2022). This evidence led the authors to speculate on the presence of toxic stress after exposure to SARS-CoV-2. ABC transporters are involved in a variety of vital bacterial functions, such as importing nutrients, biosynthetic precursors, vitamins, metals, and exporting lipids, sterols, drugs, primary and secondary metabolites, all of which affect bacterial metabolism as well as bacterial pathogenesis and virulence. In fact, the xenobiotic efflux exerted by ABC transporters is involved in the development of antimicrobial resistance (Akhtar and Turner, 2022). To note, the Biosynthesis of ansamycins pathway was also found to be specific of the COVID-19 cohort in our previous targeted-metagenomic study (Romani et al., 2022). Indeed, the 21 stool samples examined in this study were from the same sample cohort as the 16S rRNA-based metagenomics study, which had the opportunity to analyze a considerably larger number of samples (68 patients). The comparisons were conducted using a group of 21 healthy subjects, of which 12 individuals overlapped with the CTRL metagenomics cohort (95 individuals). However, the results of the two studies did not completely overlap, not only due to the difference in the sample sizes but also because the approaches were different. Metagenomics provides a comprehensive overview of the genes present in a sample’s species, with greater accuracy in taxonomy assignments and ability to infer their function. Conversely, metaproteomics yields a fluid catalog of species and their expressed proteins, revealing active genes and species with specific functions that enable a clearer understanding of functional roles and interactions within microbial communities; however, the high homology of bacterial protein sequences limits its taxonomic resolution. Indeed, especially when dealing with complex microbial populations and lacking sample-specific shotgun metagenomics-derived databases, taxonomic outcomes between the two omic approaches may vary significantly. This stems from the fact that meta-omic disciplines employ distinct analytical techniques on various molecular species (Tanca et al., 2017).

Despite the low abundance of PGs associated with DNA repair and recombination systems, it is noteworthy that these pathways were downregulated in COVID-19 pediatric patients. Furthermore, the stratification of patients based on disease severity reveals a correlation between severity and under-expression of at least one pathway (Mismatch repair) associated to DNA repair and recombination systems: the greater the severity, the greater the lack of expression, ranging from asymptomatic to mild to moderate. Thus, the study suggests that SARS-CoV-2 has a negative impact on GM by hindering the functionality of excision repair systems. This leads to a decrease of these pathways from healthy controls to individuals with asymptomatic COVID-19 and those with mild to moderate symptoms. These repair systems are activated after damage occurs to one strand of DNA. All living organisms use at least three types of excision repair systems, namely mismatch repair, BER, and NER, to correct DNA damage to ensure their survival and that of their progeny (Ha and Bhagavan, 2023). There are clear examples of how direct and indirect interactions with eukaryotic viruses can affect bacterial biology and vice versa (Neu and Mainou, 2020); in this case, it is not easy to hypothesize a mechanism of action, but it may be related to the general state of stress that bacteria face in the inflammatory environment caused by SARS-CoV-2.

In order to further investigate which microbial pathways may be associated with disease severity, we performed a correlation analysis between the KEGG pathways that we found to be differentially regulated in our patients and clinical features such as WBC and CRP. Previous retrospective studies have indicated that WBC at admission and WBC fluctuation during hospitalization were correlated with COVID-19 progression, severity and even death (Chen et al., 2020; Lv et al., 2020; Qin et al., 2020; Zhu B. et al., 2021). Furthermore, neutrophil and lymphocyte counts specifically, as well as their ratio, were also uncovered as an important prognostic markers for COVID-19 (Chen et al., 2020; Lv et al., 2020; Qin et al., 2020).

In our dataset, we found 19 KEGG pathways to be negatively correlated with WBC, while 2 were positively correlated (**Figure 7**). Among those that are negatively correlated with WBC, Fatty acid biosynthesis was also found to be enriched in our pediatric patient cohort, indicating that this metabolic pathway may be one of the mechanisms by which the pediatric GM protects the host from more severe manifestations of COVID-19. Furthermore, the significantly enriched proteins in this pathway were associated with the *Faecalibacterium* and *Ruminococcus* genera. While taxonomic inferences from proteins are predictive and not as precise as metagenomics analysis, these results are consistent with previous reports of an enrichment of *Faecalibacterium* in the GM of pediatric COVID-19 patients (Romani et al., 2022). Taken together, these results suggest that members of the *Faecalibacterium* genus may also contribute to pediatric protection against severe COVID-19 *via* modulation of fatty acid metabolism.

Severe COVID-19 has been associated with a lower lymphocyte count coupled with a higher neutrophil count compared to patients with mild or asymptomatic forms of COVID-19 (Lv et al., 2020). Therefore, we decided to also investigate whether these parameters, specifically, were correlated with the KEGG pathways found in our analysis. All 6 of the significant pathways were negatively correlated with neutrophil count (**Figure 7**). Of these, 4 were in common with a negative association with WBC, namely Valine, leucine and isoleucine biosynthesis, Thiamine metabolism, Glucosinolate biosynthesis and Nonribosomal peptide structures. Interestingly thiamine, also known as vitamin B_1_, is known to boost antibody responses, reduce pro-inflammatory cytokines and, as such, has been proposed as a potential adjuvant therapy for COVID-19 patients (Shakoor et al., 2021). In fact, critically ill COVID-19 patients who received thiamine were less likely to suffer thrombosis and were more likely to survive the disease than those who did not (Al Sulaiman et al., 2021). Furthermore, hospitalized patients with COVID-19-induced encephalopathy showed significantly improved neurological function after receiving intravenous thiamine (Branco De Oliveira et al., 2021). In this context, our results suggest that GM-derived thiamine could also contribute to protection against severe COVID-19.

Interestingly, none of the pathways that were significantly correlated with WBC and neutrophils were also significantly correlated with lymphocyte counts. Instead, we found 7 interestingly different pathways that were negatively correlated with lymphocyte counts. Since lymphocyte counts are generally negatively correlated with COVID-19 severity, KEGG pathways that are negatively correlated with lymphocyte counts should, in principle, be positively correlated with COVID-19 severity. On the one hand, some of these pathways are consistent with those that are negatively correlated with neutrophil counts. For example, while Valine, leucine and isoleucine biosynthesis was negatively correlated with neutrophil counts, Valine, leucine and isoleucine degradation was negatively correlated with lymphocyte counts (**Figure 7**). Therefore, taken together, these results suggest that GM-derived branched amino acid metabolism is associated with these clinical markers of disease severity, as their synthesis is associated with lower neutrophil counts, while their degradation is associated with higher lymphocyte counts. However, on the other hand, our results are not entirely consistent with the principle of lower lymphocyte counts being a prognostic marker for more severe forms of COVID-19. For example, Tryptophan metabolism, which was significantly negatively correlated with lymphocyte counts, was also significantly reduced in children with moderate COVID-19 compared to children with either mild or asymptomatic manifestations of the disease. It is, however, important to note that, when it comes to children, the normal ranges of lymphocyte counts varies dramatically in function of their age bracket. Therefore, when observing a wide age range of pediatric patients, lymphocyte counts may be more difficult to associate with disease, as what is a “low” count for an older child is actually a “normal” count for a younger one. Given this substantial confounding factor, further studies on a much larger cohort of patients are necessary to determine if, and how, lymphocyte counts in children correlate with COVID-19 severity, and how GM-derived metabolites may influence these clinical parameters.

Finally, we also performed this correlative analysis with serum CRP levels. CRP is a protein produced by the liver in response to systemic inflammation, and was also found to be correlated with COVID-19 severity in adults (Lv et al., 2020; Al Sulaiman et al., 2021) and with multisystemic inflammatory syndrome in children (MIS-C) (Ozsurekci et al., 2021). In our dataset, Glycosphingolipid biosynthesis KEGG pathway was negatively correlated with serum CRP levels (**Figure 7**). Excessive Glycosphingolipid biosynthesis can precipitate the generation of pro-inflammatory cytokines and lead to tissue damage in general, and in COVID-19 in particular (Trivedi et al., 2022). These results suggest that the GM too can modulate the synthesis of glycosphingolipids in response to COVID-19, and that this modulation can in turn influence their pro-inflammatory properties.

Among the differentially expressed human PGs, we found a down-expression of the Fibrinogen γ-chain. Previous studies have shown an increased risk of prothrombotic coagulation abnormalities in COVID-19 patients, with higher disease severity and increased mortality rates associated with elevated blood levels of D-dimer, fibrinogen, and platelets (Cunningham et al., 2022; Shama et al., 2023). Our counter-evidence was based on fecal samples in a cohort of pediatric patients at the onset of COVID-19 who did not MIS-C. Therefore, this outcome can be attributed to the presence of a more efficient systemic inflammatory response that counters SARS-CoV-2 action, resulting in milder symptoms compared to adults.

Ideally, this study should have been conducted on a larger sample size. As a consequence, second limitation arose from the fact that we were not able to stratify patients based on different age range knowing that GM significantly changes during the time frame of 1-16 years. Therefore it will be crucial to validate the results on a larger cohort while also accounting for variations within age groups to better understand the protective role played by GM in protection against COVID-19.

To note, studies on the correlation between the GM and respiratory diseases in children are still scarce and the direction of this interaction remains unclear: do fluctuations (due to environmental, dietary, or genetic factors) in the GM increase or decrease the risk of respiratory tract infections, or do respiratory tract infections cause alterations in the gut microbiota? (Zhu W. et al., 2021). What is certain is that the microbiota has an impact on the pediatric immune system and it has been demonstrated that the GM plays a critical role in safeguarding against bacterial and viral respiratory infections by steering the innate and adaptive immune response (Wiertsema et al., 2021). As example, variations in young children GM composition were observed in relation to respiratory syncytial virus disease (RSV) severity (Harding et al., 2020), although it remains unclear whether these alterations were a cause or a result of RSV infection. Anyway, it has been shown that the modulation of intestinal bacteria can be an effective clinical treatment to prevent severe symptoms of respiratory diseases such as asthma (Liu A. et al., 2021).

Moreover, our observations of an overexpression of PGs associated with *Faecalibacterium* in COVID-19 samples and the hypothesis that this may contribute, among other factors, to the reduced severity of disease in children are consistent with the findings in adult patients. Numerous studies have documented the scarcity of this genus that produces butyrate in COVID-19; its presence, when combined with *Roseburia*, successfully differentiated between critical and mild forms of the illness (Reinold et al., 2021). Indeed, a recent systematic review has brought to light a distinctive microbial composition in adult COVID-19 patients: there is a reduction in SCFAs-producing bacteria, like *Faecalibacterium*, within their GM, which persists even after recovery (Simadibrata et al., 2023). Therefore, targeting GM as a therapeutic option or considering it as adjuvant therapy following SARS-CoV-2 infection is highly attractive.

Fecal microbiota transplantation (FTM) can then be considered (Wang et al., 2022). One study has been already been published demonstrating how targeting FMT has favorable effects on the patients’ GM and immune system after SARS-CoV-2 infection (Liu F. et al., 2021). Indeed, FMT may also provide benefits to patients experiencing post-acute COVID-19 syndrome (PACS, or long COVID-19), which is characterized by long-term complications and/or persistent symptoms after contracting COVID-19. GM of adult patients with PACS were described by being dysbiotic with lower levels of butyrate-producing bacteria, including *F. prausnitzii* (Liu et al., 2022; Norouzi Masir and Shirvaliloo, 2022). It is worth noting that adult patients, who previously were recovered and later suffered from long COVID, experienced GM dysbiosis even one year after being discharged from medical care (Zhang et al., 2023). Their GM showed reduced bacterial diversities and lower relative abundance of SCFAs-producing salutary symbionts, such as *Eubacterium_hallii*_group, *Subdoligranulum*, *Ruminococcus*, *Dorea*, *Coprococcus*, and *Eubacterium_ventriosum*_group (Zhang et al., 2023).

Taken together, our data obtained by the metaproteomics approach showed, as the GM of pediatric patients is modulated by COVID-19 infection, it can attenuate disease severity through multiple processes, such as modulation of various metabolic pathways, antibiotic resistance and virulence mechanisms. Some of the functional profiles enriched in children are in line with previous studies into promising adjuvant therapies for severe COVID-19 in adults. This further underscores the relevance of the pediatric GM in uncovering clinically relevant therapeutic strategies against SARS CoV-2 infection. Our findings have the potential to reveal additional, novel therapeutic strategies for future clinical studies.

## Data availability statement

The datasets presented in this study can be found in an online repository. The name of the repository and accession number can be found below: MASSIVE (https://massive.ucsd.edu/ProteoSAFe/static/massive.jsp), MSV000092639.

## Ethics statement

This study involving humans was approved by Bambino Gesù Children’s Hospital Ethics Committee. (Protocol code 2083_OPBG_2020) and was conducted in accordance with the local legislation and institutional requirements. Written informed consent for participation in this study was provided by the participants’ legal guardians/next of kin.

## Author contributions

VM: Data curation, Formal Analysis, Investigation, Methodology, Visualization, Writing – original draft, Writing – review & editing. SLM: Investigation, Methodology, Writing – review & editing. CM: Formal Analysis, Investigation, Methodology, Visualization, Writing – review & editing. AP: Investigation, Writing – original draft, Writing – review & editing. FR: Formal Analysis, Investigation, Writing – original draft. SP: Investigation, Writing – review & editing. FDC: Investigation, Writing – review & editing. PV: Investigation, Writing – review & editing. LR: Data curation, Investigation, Writing – review & editing. AC: Investigation, Writing – review & editing. PP: Investigation, Writing – review & editing. LP: Conceptualization, Funding acquisition, Investigation, Methodology, Supervision, Writing – review & editing.

## Group members of CACTUS Study Team

Stefania Bernardi, MD, Francesca Calò Carducci, MD, Caterina Cancrini, MD, PhD, Sara Chiurchiù, MD, Marta Ciofi degli Atti, MD, Nicola Cotugno, MD, Laura Cursi, MD, Renato Cutrera, MD, Carmen D’Amore, MD, Patrizia D’Argenio, MD, Maria A. De Ioris, MD, Maia De Luca, MD, Carlo Federico Perno, Prof., Andrea Finocchi, MD, PhD, Laura Lancella, MD, Giulia Linardos, PhD, Emma Concetta Manno MD, Elena Morrocchi, PhD, Paola Pansa, MD, Paolo Rossi, Prof., Libera Sessa, PhD, Alberto Villani, Prof., Paola Zangari, MD.

## Supplementary material

The Supplementary Files for this article can be found online at: https://www.frontiersin.org/articles/10.3389/fcimb.2023.1327889/full#supplementary-material
