# Supplementary material for: The metaproteome of the gut microbiota in pediatric patients affected by COVID-19
Source: Front Cell Infect Microbiol. 2023 Dec 22;13:1327889. doi: 10.3389/fcimb.2023.1327889 (PMC10766818; doi:10.3389/fcimb.2023.1327889)
Supplement: Supplementary file 1 [file DataSheet_1.zip › SupplementaryFigures&Data.docx]

Supplementary Material

# Supplementary Data

## Supplementary Files

**Supplementary File 1.** Metadata associated to COVID-19 patients and healthy control subjects.

**Supplementary File 2.** List of differentially expressed bacteria and human Protein Groups from faecal samples of COVID-19 patients and age-matched healthy subjects (CTRLs) accompanied by functional, taxonomic annotations, and differentially expressed bacterial KEGG pathways.

**Supplementary File 3.** Results of DAVID enrichment terms of human Protein Groups.

**Supplementary File 4.** List of differentially expressed bacteria and human Protein Groups of the GM found in COVID-19 patients categorized by disease severity as “asymptomatic”, “mild”, or “moderate”, accompanied by functional, taxonomic annotations, and differentially expressed bacterial KEGG pathways.

# Supplementary Figures

## Supplementary Figures

**
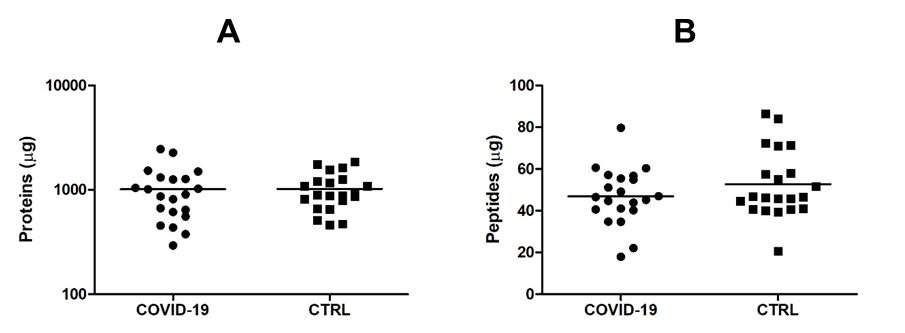
**

**Supplementary Figure 1.** Scatter plot graphs of quantified extracted proteins (panel A), from stool samples of COVID-19 patients and age-matched healthy subjects (CTRLs), as well as purified peptides (panel B). Mean values are evidenced as back bar.


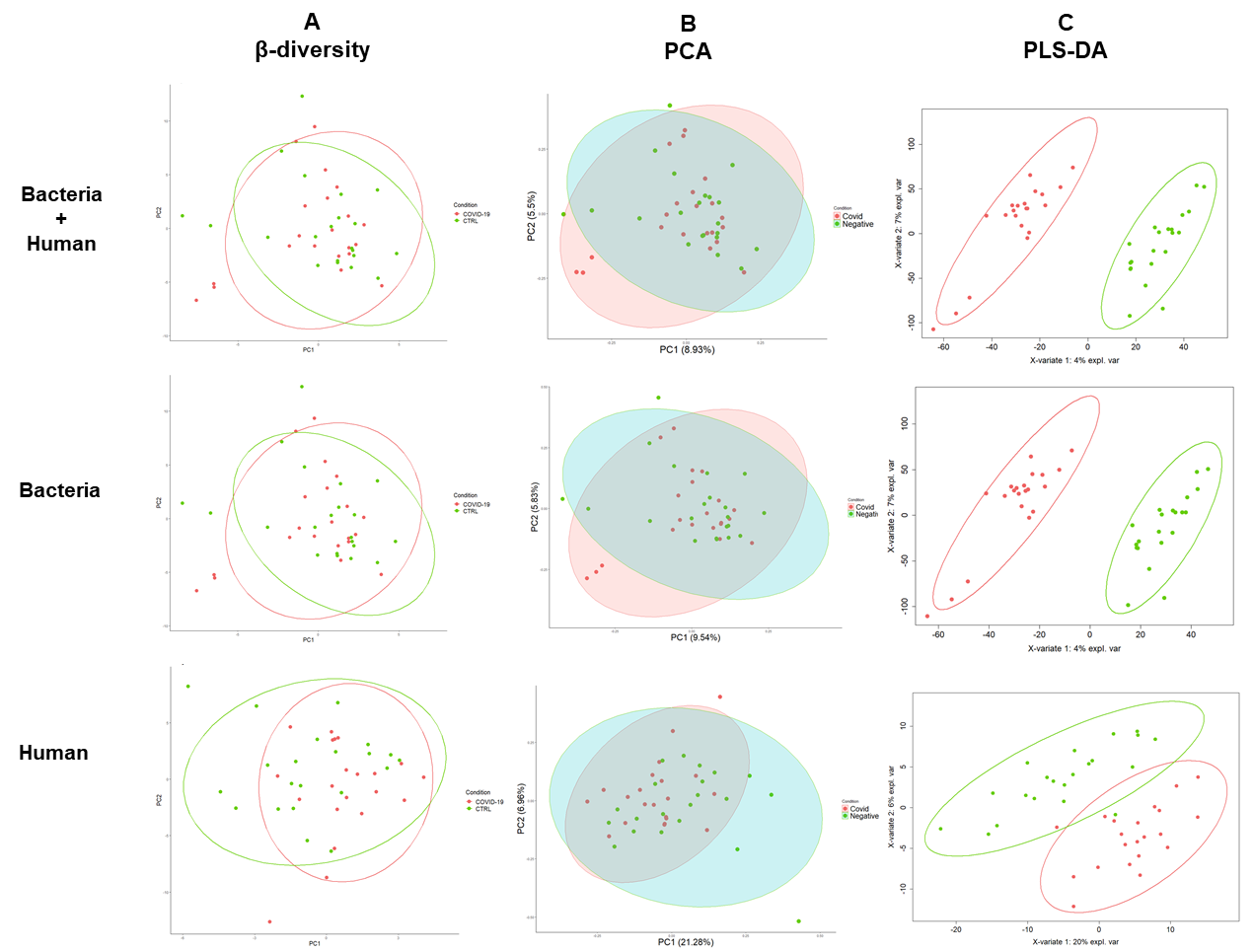


**Supplementary Figure 2.** Analyses of quantified protein groups (PGs) from stool samples of patients (COVID-19, red colour) and age-matched healthy subjects (CTRL, green colour). The dissimilarity between COVID-19 and CTRL groups was quantified using unsupervised Bray-Curtis β-diversity analysis, based on their protein content (Panel A). Through the PERMANOVA test, the human PGs’ dataset alone showed a statistically significant (*p*-value = 0.03) divergence. Unsupervised Principal Component Analysis (PCA) displayed a slight separation amongst the two groups (Panel B). The Supervised Partial Least Squares-Discriminant Analysis (PLS-DA) model showed significance solely when examining human PGs alone (Q2 = 0.502, *p*-value = 0.01) (Panel C).

**
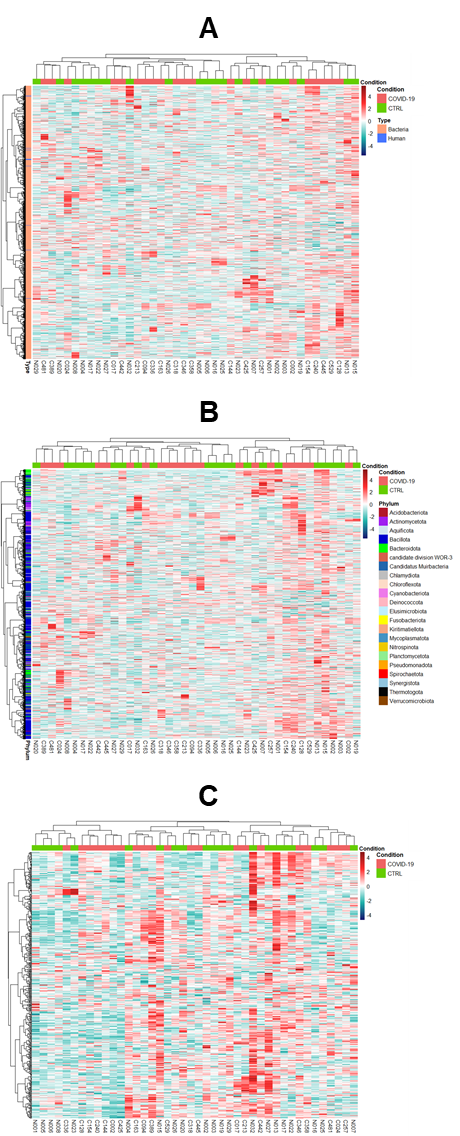
**

**Supplementary Figure 3.** Cluster analyses of identified protein groups (PGs) from stool samples of patients (COVID-19, red colour) and age-matched healthy subjects (CTRL, green colour). A heat map based on LFQ PGs’ intensity abundances and subject to a z-score transformation was used to visualize color-coded hierarchical cluster analysis. The analysis was performed for all PGs (Panel A), bacterial PGs (Panel B), and human PGs (Panel C). The dendrogram depicted above the heat map indicates that there was no similarity observed among samples within either of the two groups.


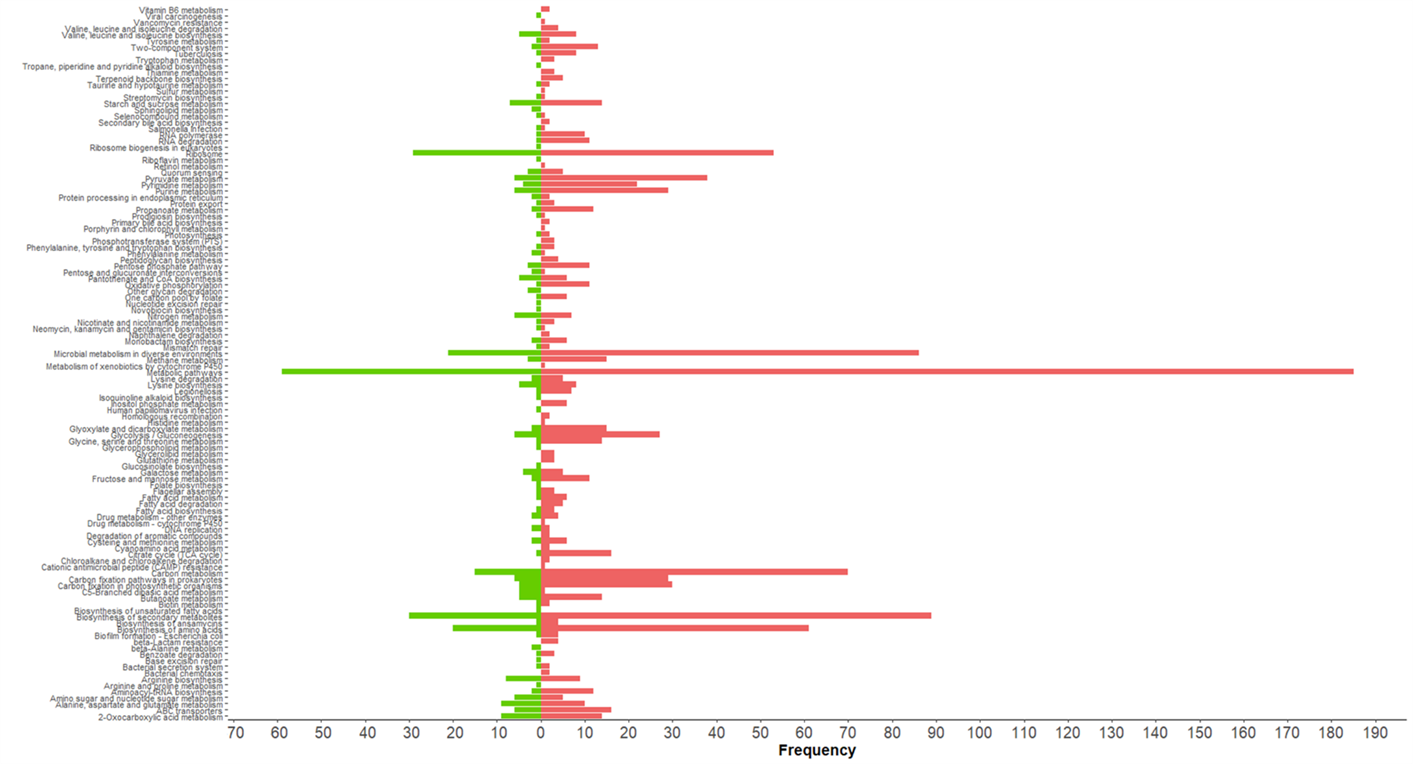


**Supplementary Figure 4**. Graphical representation of the 114 KEGG pathways associated to the 675 differentially expressed bacteria protein groups (PGs) in COVID-19 and CTRLs. The bars display the frequency of the number of differentially expressed bacteria PGs associated with the pathway annotation. Color codes red and green represent PGs over and under-expression in COVID-19, respectively.


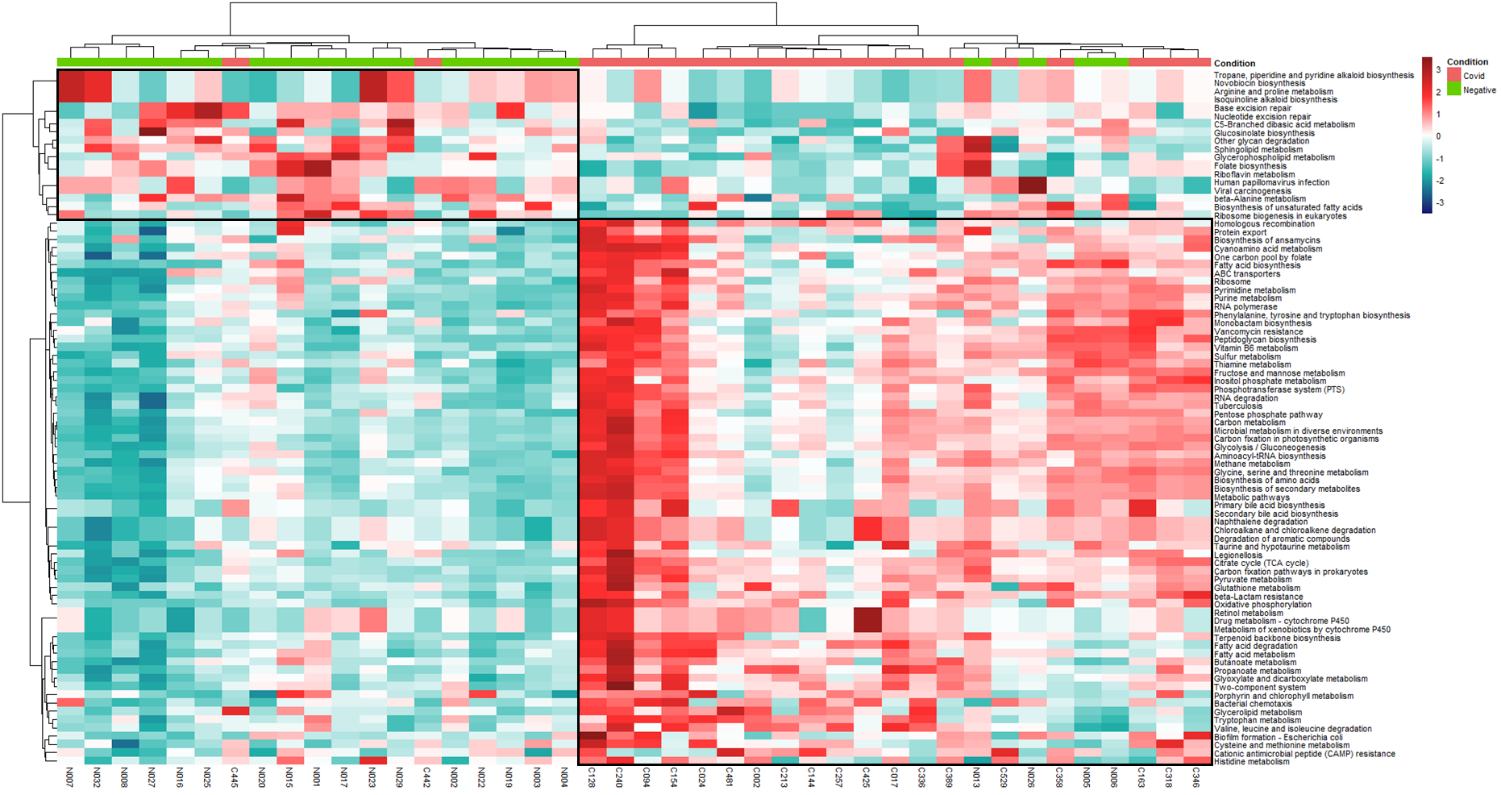


**Supplementary Figure 5.** Cluster analyses were conducted on 84 differentially expressed KEGG pathways that were associated with the 675 differentially expressed bacterial protein groups (PGs) found in stool samples from patients with COVID-19 (indicated in red) and age-matched healthy controls (CTRL, indicated in green). The mean intensity abundance of PGs with the same KEGG pathways for each sample was calculated. Statistically significant pathways were then identified by computing a *t*-test and adjusted *p*-value for false discovery rate (using Benjamini-Hochberg correction for independent or positively correlated tests) when comparing COVID-19 and CTRL groups. A heat map based on the data was created after a z-score transformation. Bolded data evidenced the group of over- (on the right lower site) and under-expressed (on the left upper site) KEGG pathway in COVID-19 patients.


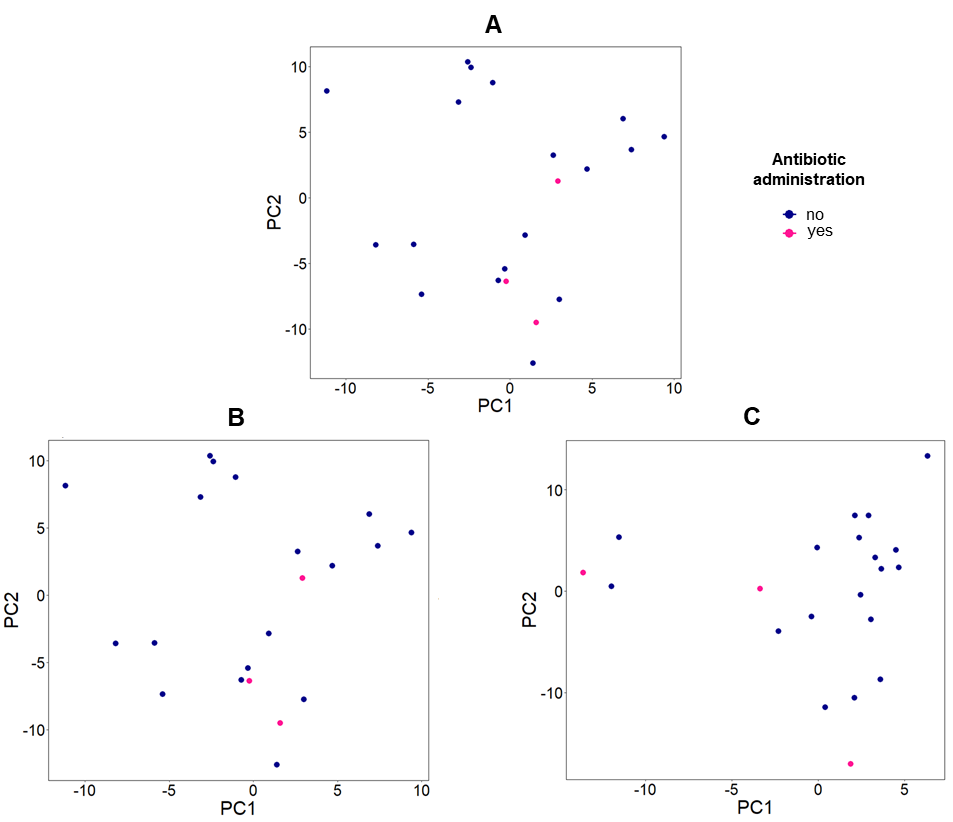


**Supplementary Figure 6.** Bray-Curtis β-diversity analysis of quantified protein groups (PGs) from stool samples of COVID-19 patients categorized according to their antibiotic administration prior to sample collection (“no” = blue colour and “yes” = pink colour). The analysis was performed on all PGs (bacterial and human, Panel A), as well as on separate datasets of bacterial PGs (Panel B) and human PGs (Panel C).
